# Supplementary material for: Robust projection of East Asian summer monsoon rainfall based on dynamical modes of variability
Source: Nat Commun. 2023 Jun 29;14:3856. doi: 10.1038/s41467-023-39460-y (PMC10310711; doi:10.1038/s41467-023-39460-y)
Supplement: Supplementary file 1 — Supplementary Information [file 41467_2023_39460_MOESM1_ESM.pdf]

Robust projection of East Asian summer monsoon rainfall through the lens of the dynamical modes of variability

Daokai Xue[1*], Jian Lu[2*], and L. Ruby Leung[2], Haiyan Teng[3], Fengfei Song[4,5], Tianjun Zhou[6], and Yaocun Zhang[1]

[1]School of Atmospheric Sciences, Nanjing University, Nanjing, China

[2]Atmospheric Sciences and Global Change Division, Pacific Northwest National Laboratory, Richland, WA, USA

[3]Climate and Ecosystem Sciences Division, Lawrence Berkeley National Laboratory, Berkeley, CA, USA

[4]Frontier Science Center for Deep Ocean Multispheres and Earth System and Physical Oceanography Laboratory, Ocean University of China, Qingdao, China

[5]Laoshan Laboratory, Qingdao, China

[6]State Key Laboratory of Numerical Modeling for Atmospheric Sciences and Geophysical Fluid Dynamics, Institute of Atmospheric Physics, Chinese Academy of Sciences, Being, China

Correspondence should be directed to:

Jian Lu (jian.lu@pnnl.gov) and Daokai Xue (dkxue@nju.edu.cn)

Contents of this file

Figures S1 to S8
Tables S1

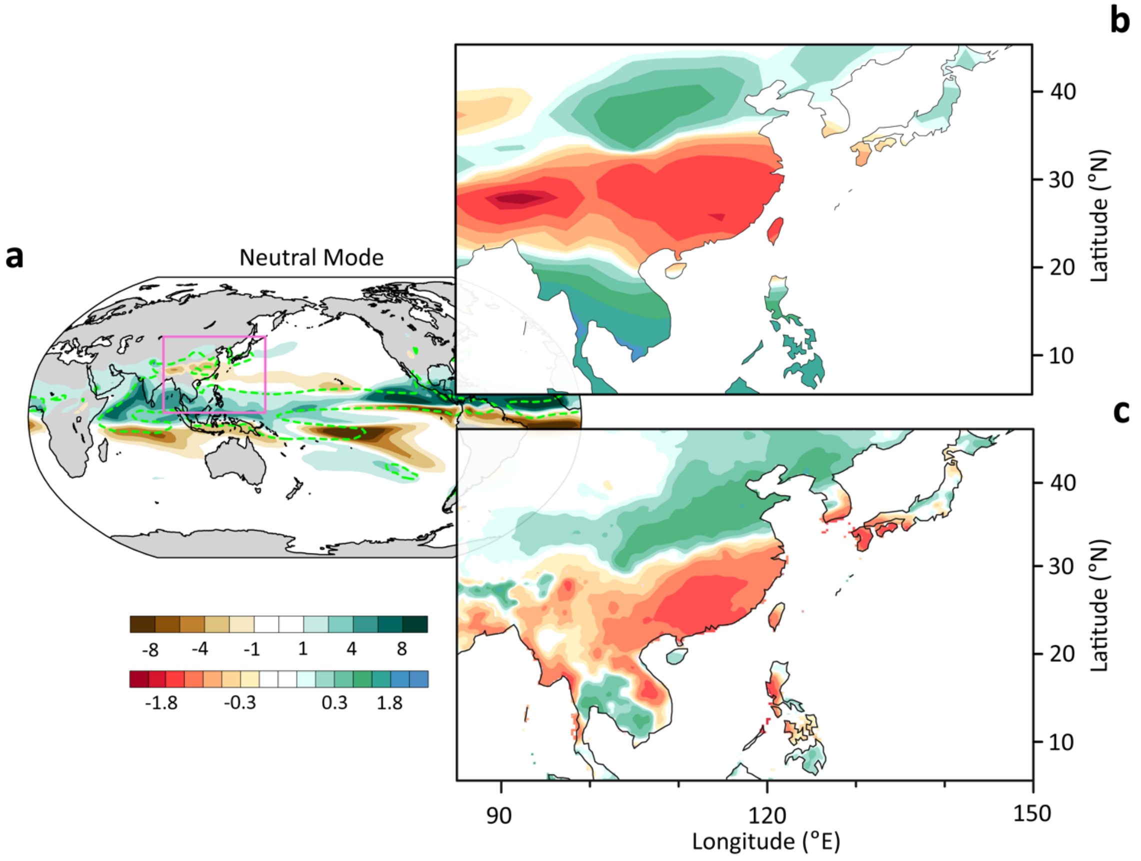

Figure S1 Agreement between NM2 and EOF2 of East Asian summer monsoon rainfall. (a) The 2nd neutral mode (NM2) of the global June-July-August (JJA) precipitation extracted from the precipitation linear response function (LRF) (unit is mm day^{-1}). The green dashed contours indicate the 6 mm day^{-1} isoline of the JJA summer rainfall climatology. (b) a close-up of NM2 over East Asia to facilitate the comparison with the observed second empirical orthogonal function (EOF2) pattern of summer rainfall over the same region shown in (c).

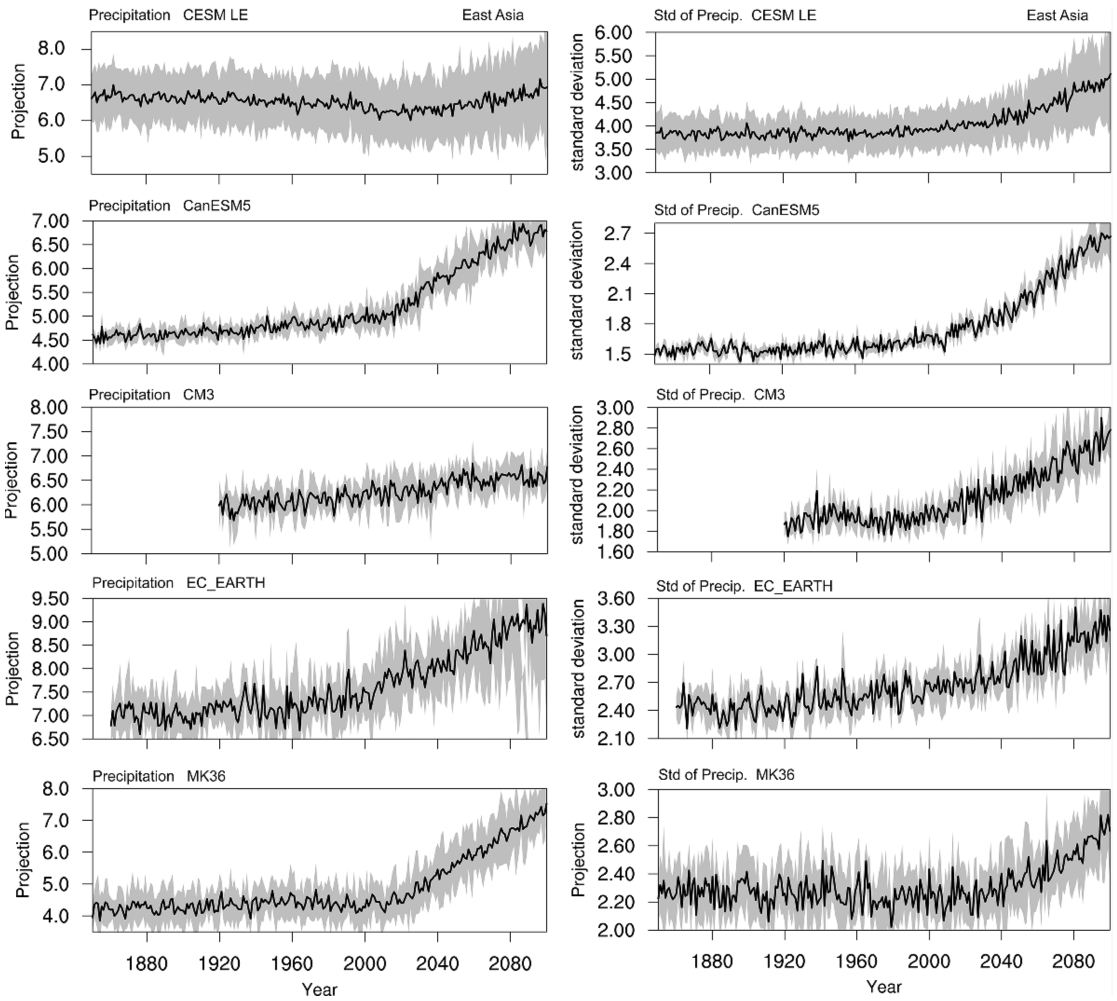

Figure S2. **The evolution of the NM2 projections in CESM-SMILEs.** Left panels: Projections of JJA seasonal mean precipitation onto NM2 over East Asia, or NM2 index. Right panels: The std of summer daily NM2 index. Solid line in left (right) panels in each panel is the MME mean projection (ensemble mean of the std of the daily projection). Shading indicates the averaged one std of the ensemble members of each individual ensemble.

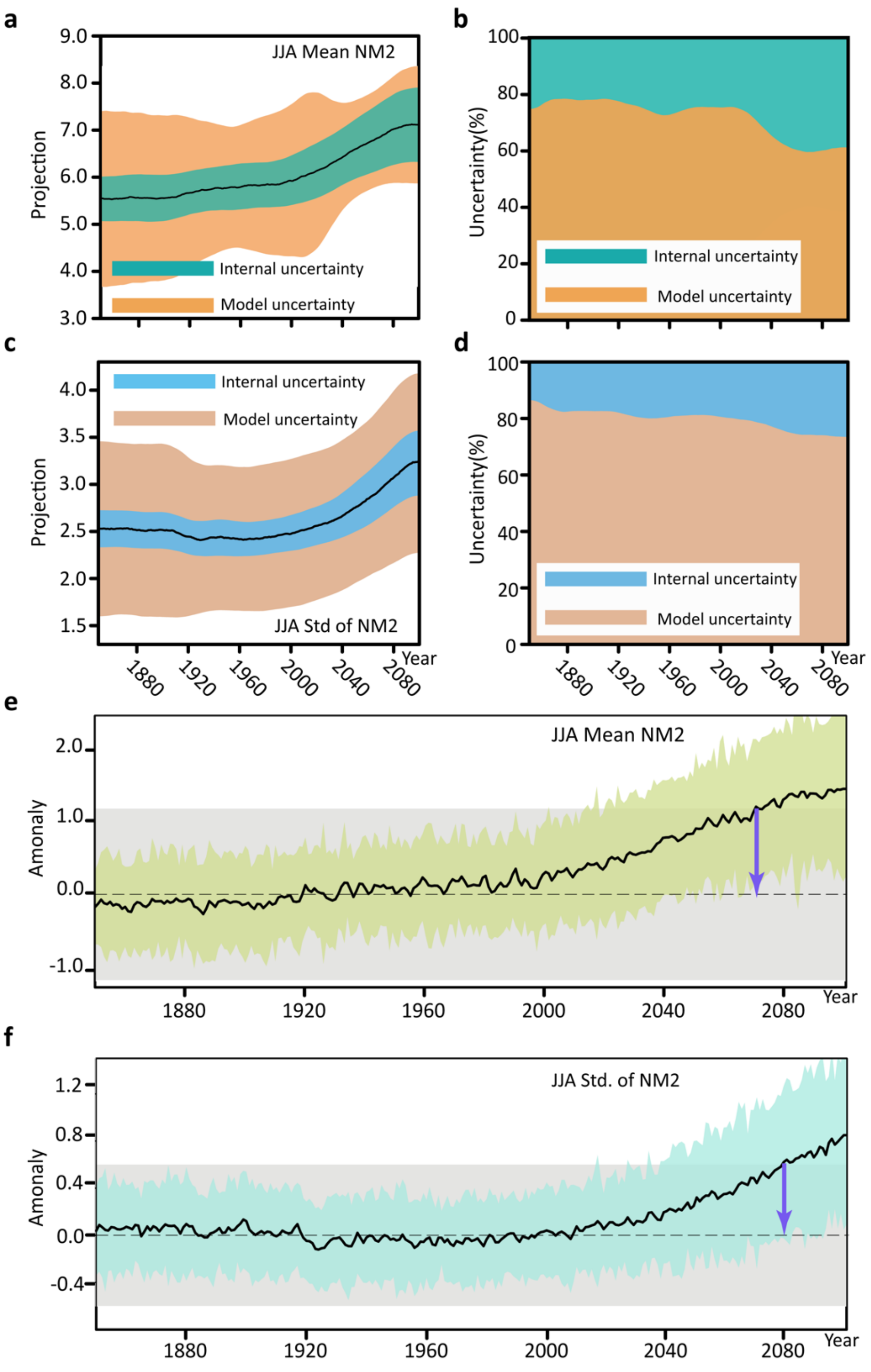

Figure S3. The evolution of uncertainty components and ToE of the forced NM2 response. (a) the evolution of the multi-model ensemble mean (MMEM) NM2 index and its internal and model uncertainties represented by two std (i.e., $2\sqrt{\bar{\bar{I}}_t}$ and $2\sqrt{M_t}$, respectively) on both sides of the MMEM; (b) fractional contribution to the total uncertainty for the two uncertainty sources; (c) same as (a) but for the std of the NM2 index; (d) same as (b) but for the std of the NM2

index; Note that a 10-year running mean has been applied to these time series. (e) Time evolution of the forced response of NM2 index (black line) with respective to the historical (1850 to 2010) mean. The light green shading indicates the upper and lower bounds of the grand ensemble. The blue and red arrows demarcate the ToE based on two types of definition (see text for details). (f) same as (e) but for the std of the NM2 index. In both (e) and (f), grey shading indicates the upper and lower bounds of the grand internal variability during the historical period (1850-2010).

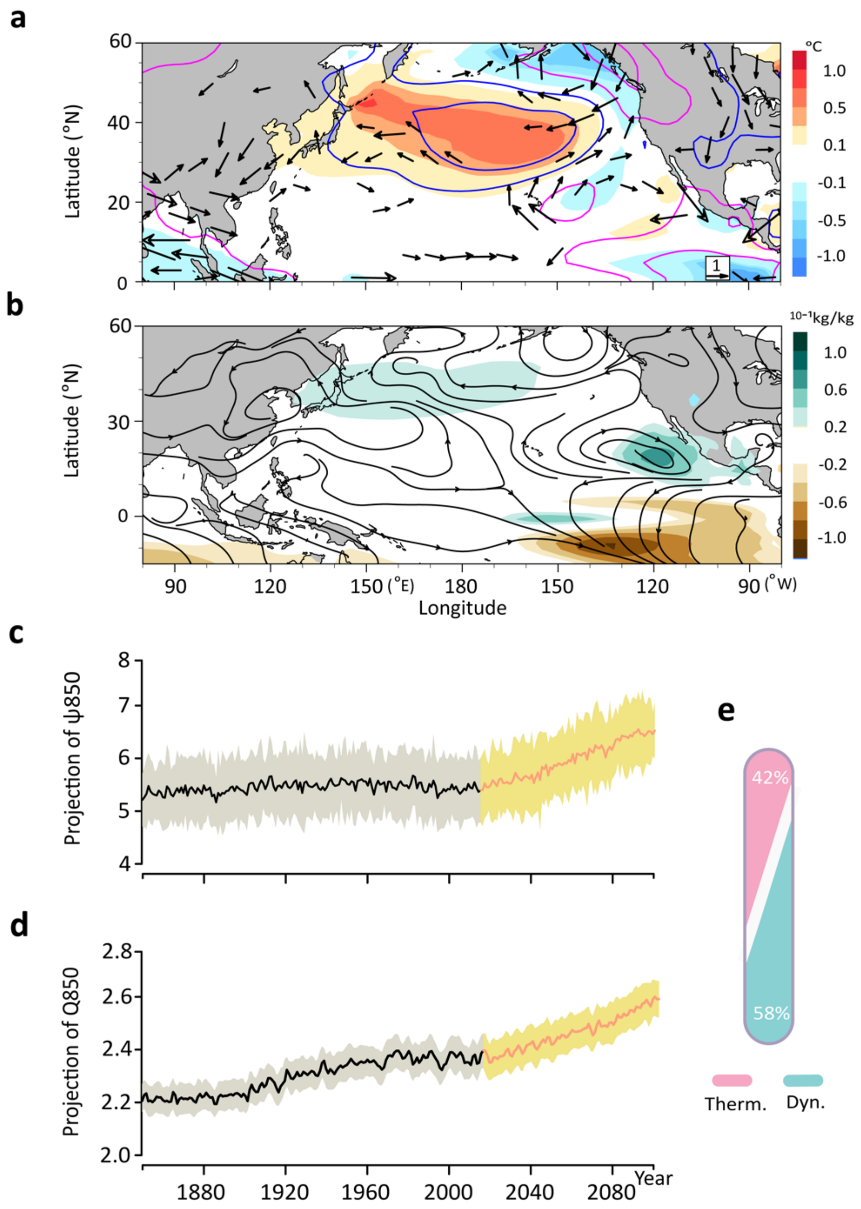

Figure S4. The associated dynamic patterns of NM2. (a) The NM2-associated patterns in sea surface temperature (shading), 850 hPa wind (vectors, ms^{-1}), and 500hPa geopotential height (contours, gpm). (b) The NM2-associated patterns in $Q850$ (shading) and streamline (arrowed lines). (c) The time series of the projection of $\psi850$ onto the NM2-associated pattern. The shading indicates the two std of the internal variability of the grand ensemble. (d) Same as (c) but for the time series of the projection of $Q850$ onto the NM2-associated pattern. (e) The fractional contributions to the trend during the projection period from the dynamic (light blue) and thermodynamic (pink) components in CESM-SMILEs.

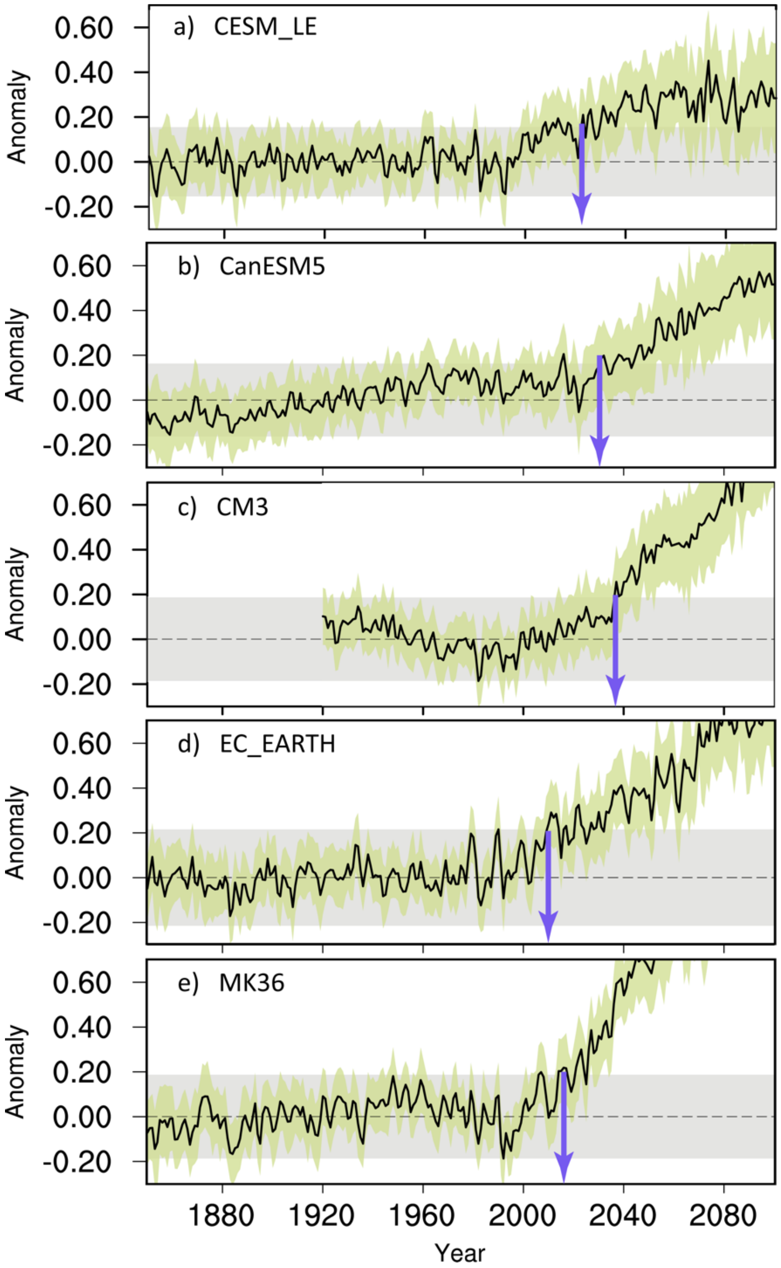

Figure S5. Time evolution of the forced response of NM2 index and the associated ToEs. The light green shading indicates the upper and lower bounds of the multi-ensembles for corresponding model. The grey shading indicates the upper and lower bounds of the grand internal variability during the historical period (1850-2010). The blue arrow indicates the ToE as the "first year of ensemble mean out".

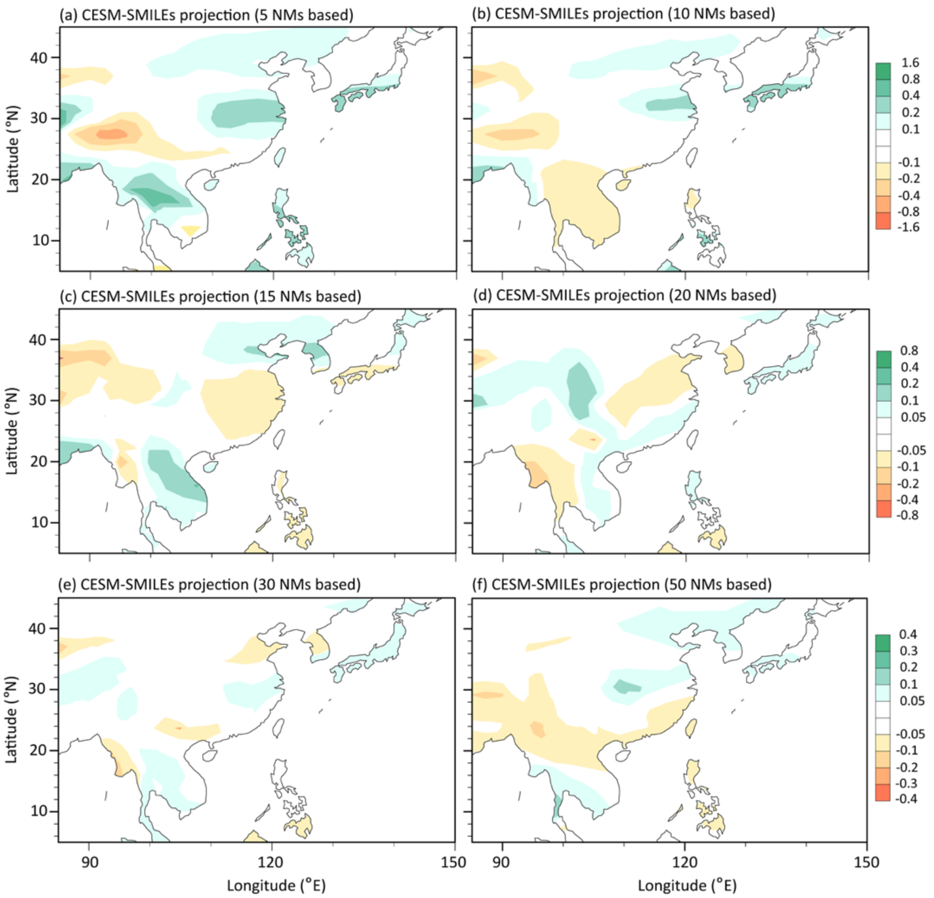

Figure S6. The NM-based multi-model projections with increasing number of NMs included. When more and more NMs are used in the NM-based projections, the resultant pattern can gradually approach the projection using conventional multi-model ensemble mean (cf. Figure 5b).

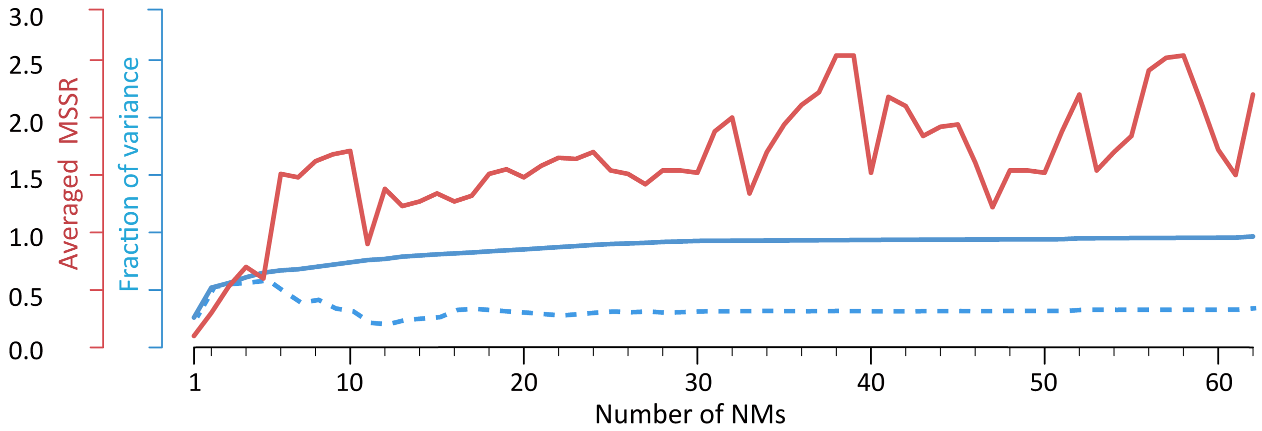

Figure S7 A trade-off between number of NMs and confident projection with high model spread-to-signal ratio (MSSR). Blue line: The fraction of the variance of the CESM-SMILEs grand ensemble mean precipitation change explained by the NM-based projections as the number of NMs included increases (y value=1 indicates 100% variance explained). Blue dashed line: The fraction of variance of the CESM-LENS2 ensemble mean precipitation change explained by the NM-based projections using the SMILEs ensembles. Red line: The MSSR (see Methods for definition) for each NM-based projection computed as the ratio of the variance of the inter-model spread to the variance of the grand ensemble mean signal (y value=1 means noise level is as high as the signal).

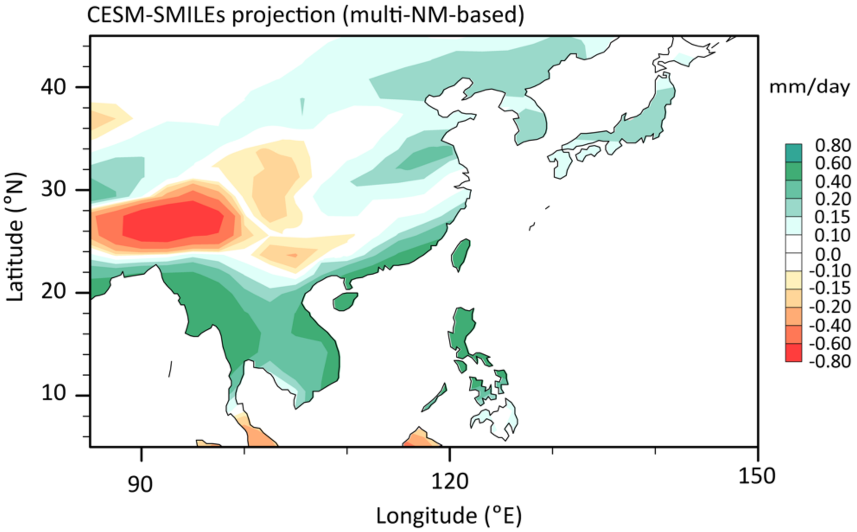

Figure S8. Multi-NM-based projection of the JJA precipitation. Similar to Figure 5a, but using NM1, NM2, NM3, NM5, NM6, NM7, NM9 and NM10. See Methods for the criteria for choosing the NMs for projection. The variance of this projected change of precipitation is 78 $mm^2\ day^{-2}$, in contrast to 52 $mm^2\ day^{-2}$ based on the first two NMs (Figure 5a).

Mode2 / Mode1	CanESM5	CM3	EC_EARTH	MK36	CESM2	Obs.
CanESM5	1	0.50*	0.41*	0.18	0.44*	0.32*
CM3	0.61*	1	0.29	0.27	0.66*	0.52*
EC_EARTH	0.42*	0.66*	1	0.32*	0.79*	0.81*
MK36	0.54*	0.51*	0.20	1	0.51*	0.70*
CESM2	0.40*	0.69*	0.27	0.30	1	0.48*
Obs.	0.34*	0.49*	0.43*	0.56*	0.60*	1

Table S1. Pattern correlations among the EOF1s and EOF2 from CESM-SMILEs and the observation. The pattern correlation is computed between each pair of the EOF patterns from the CESM-SMILEs models and the APHRODITE observation. Asterisk indicates the significance of the pattern correlation at 95% confidence level. The confidence level is estimated against the distribution of the pattern correlations computed from 10,000 pairs of samples of JJA seasonal precipitation, each being taken randomly from the model-model pair or model-observation pair being investigated.
